# Total body irradiation as part of conditioning regimens in childhood leukemia—long-term outcome, toxicity, and secondary malignancies

**DOI:** 10.1007/s00066-021-01810-4

**Published:** 2021-07-19

**Authors:** Arne Gruen, Sebastian Exner, Jörn-Sven Kühl, Arend von Stackelberg, Volker Budach, Carmen Stromberger, Dirk Boehmer

**Affiliations:** 1 Department for Radiation Oncology and Radiotherapy, Charité—Universitätsmedizin Berlin, corporate member of Freie Universität Berlin, Humboldt-Universität zu Berlin, and Berlin Institute of Health, Campus Virchow-Clinic, Augustenburger Platz 1, 13353 Berlin, Germany; 2 Strahlenzentrum Hamburg MVZ, Langenhorner Chaussee 369, 22419 Hamburg, Germany; 3 Department for Pediatric Oncology, Hematology and Hemostaseology, University Clinic Leipzig, Liebigstraße 22, Haus 7, 04103 Leipzig, Germany; 4 Department for Pediatric Hematology, Oncology and Stem Cell Transplantation, Charité—Universitätsmedizin Berlin, corporate member of Freie Universität Berlin, Humboldt-Universität zu Berlin, and Berlin Institute of Health, Berlin, Germany

**Keywords:** Survival, Late adverse events, Radiation induced cancer, Pediatric, Cancer of the blood

## Abstract

**Background:**

Total body irradiation (TBI) is an established part of conditioning regimens prior to stem cell transplantation in childhood leukemia but is associated with long-term toxicity. We retrospectively analyzed survival, long-term toxicity, and secondary malignancies in a pooled cohort of pediatric patients (pts.) treated with the same TBI regimen.

**Methods:**

Analyzed were 109 pts. treated between September 1996 and November 2015. Conditioning treatment according to EBMT guidelines and the ALL SCTped 2012 FORUM trial consisted of chemotherapy (CT) and TBI with 2 Gy b.i.d. on 3 consecutive days to a total dose of 12 Gy. Median follow-up was 97.9 months (2–228 months).

**Results:**

Overall survival (OS) in our cohort at 2, 5, and 10 years was 86.1, 75.5, and 63.0%, respectively. Median survival was not reached. Long-term toxicity developed in 47 pts. After chronically abnormal liver and kidney parameters in 31 and 7 pts., respectively, growth retardation was the most frequent finding as seen in 13 pts. Secondary malignancies were rare (*n* = 3).

**Conclusion:**

TBI-containing conditioning regimens in pediatric stem cell transplantation (SCT) are highly effective. Efforts to replace TBI- with CT-containing regimens have only been successful in subgroups of pts. Although we could show long-term toxicity in 43% of pts., overall survival was 63% at 10 years. Still, long-term effects such as growth retardation can permanently impact the pts.’ quality of life and functioning. Along with new substances, efforts should be undertaken to optimize TBI techniques and accompany the treatment by systematic follow-up programs beyond 5 years to improve detection of rare events.

## Introduction

TBI reduces the number of leukemic cells and prevents graft rejection in SCT. A randomized study in pediatric pts. showed that TBI-containing induction therapy yielded better survival than CT induction alone [1]. This could be corroborated in a 2011 meta-analysis [2]. Recently, the experimental arm of the randomized ALL SCTped 2012 FORUM trial (NCT01949129) comparing TBI/VP16 conditioning versus CTpoly-CT (either busulfan/fludarabine/thiotepa or treosulfan/fludarabine/thiotepa) has been closed prematurely due to a steep increase in events when omitting TBI. However, TBI-containing regimens are associated with long-term morbidity like infertility or growth retardation, although exact rates remain unclear [3]. To help better understand the long-term effects of combined treatment approaches, we reviewed a large cohort of pediatric pts. who received the same TBI regimen (from September 1996 to November 2015) with respect to survival, long-term toxicity, and secondary malignancies.

## Methods

A total of 201 TBI pts. were treated between September 1996 and November 2015 at the Charité Department of Radiation Oncology (median follow-up 97.9 months, range 2‑228 months). After excluding adult pts. and pts. with different regimens, 109 pts. remained for analysis. Inclusion criteria were age < 19 years, primary or recurrent leukemia, 12 Gy TBI with single doses of 2 Gy. Conditioning was according to EBMT guidelines [4] and the ALL SCTped 2012 FORUM trial (NCT01949129). Prior whole-brain irradiation had been received by 45 pts. (WBRT; median 18.4 Gy, range 12–24 Gy). TBI was initially applied on a cobalt-60 machine (1996–2005) and subsequently (2006–2015) on a linear accelerator (linac) with a translation couch and the linac in 0° position. The patient is thus transported with constant velocity through the beam, first in prone then in supine position. This technique enlarges the field size, improves the photon fluence uniformity, and reduces the depth-dose inhomogeneity [5]. Very young pts. were treated under general anesthesia (*n* = 5). Data were gathered from the internal patient documentation system (SAP, SAP, Walldorf, Germany), OPS and ICD codes and from pts.’ files or through direct contact with either treating physicians, pts., or caregivers, and eventually by contacting the residents’ registration office. The data were analyzed using IBM SPSS version 22.0 (SPSS, IBM, Armonk, NY, USA). Graphical representation of survival analyses was performed using the Kaplan–Meier method. The Charité ethics board approved this study (EA2/112/21). The informed consent requirement was waived. The research complied with the Declaration of Helsinki.

## Results

**Fig. 1:**
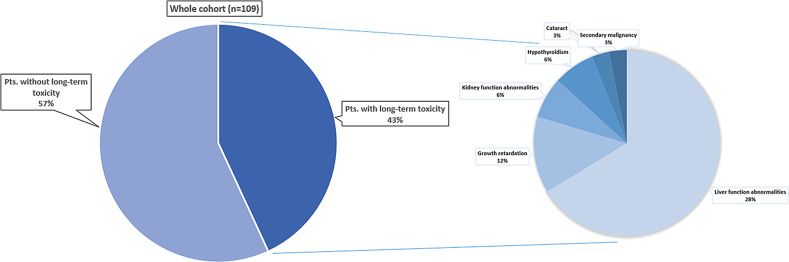
Long-term toxicity. Out of the whole cohort of 109 pts., long-term toxicity was found in 47 (43%), with chronically abnormal liver function tests, growth retardation, abnormal kidney function test, and hypothyroidism being the most frequent. Secondary malignancy and cataracts were found in only 3 pts. each. Since pts. could show more than one late effect, the numbers overlap. The percentages refer to the whole cohort of 109 pts. *Pts.* patients

**Fig. 2:**
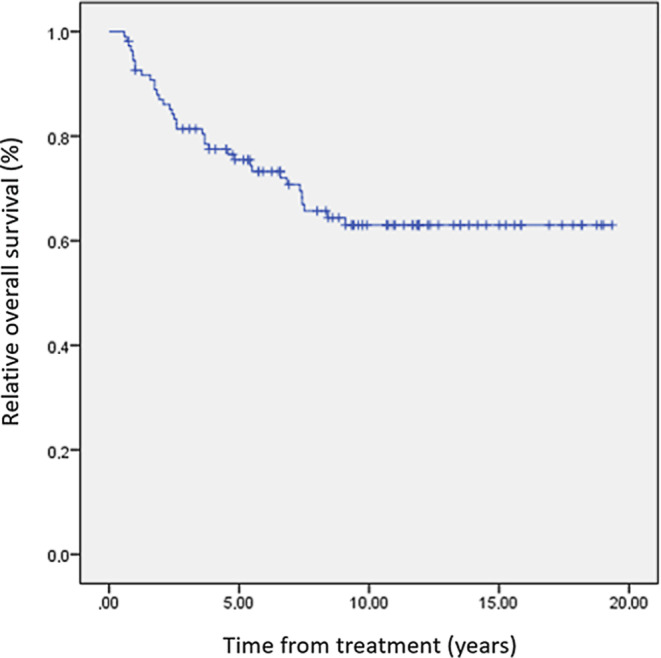
Overall survival (OS) in our cohort at 2, 5, and 10 years was 86.1, 75.5, and 63.0%, respectively. Median survival was not reached. Patients treated in CR2 (2nd complete remission) showed similar OS data at 2, 5, and 10 years with 97.8, 88.0, and 65.7%, respectively

For pts.’ characteristics see Table 1. OS in our cohort at 2, 5, and 10 years was 86.1, 75.5, and 63.0%, respectively. Median survival was not reached (Fig. 1). Late effects occurred in 47 (43%) pts. (Fig. 2). Cataracts were found in 3 pts., although the rate of visual impairment was higher. One pt. who had prior 24 Gy whole brain irradiation (WBRT) later also developed bilateral sicca syndrome. Six pts. had bone/cartilage damage. One patient (17.6 years) developed severe osteoporosis 5 months after TBI, while two showed femoral/femoral head osteonecrosis 4–5 years after TBI. Two pts. had osteopenia-related fractured vertebral bodies after 4 and 8 years. All pts. had ample exposure to steroids. Growth deficiency (GD) was diagnosed in 13 pts. (median age at treatment 9.4, range 3.4–18.7 years). Four of these pts. had prior WBRT. Hypothyroidism occurred in 7 pts. (12.5%). Two of these had prior WBRT. Abnormal liver function tests were seen in 31 pts. (40.3%; male-to-female ratio 6:25). Eight of them died within the FU period. Dyslipidemia was seen in 4 pts. All of them had pathologic serum triglycerides. Secondary malignancies were diagnosed in 3 pts. (2.8%) after a median latency period of 75.6 months (range 26–120 months). One patient developed Hodgkin’s disease (HD) 4 years after treatment on the GM-ALL 96 protocol. The patient had cobalt-60 TBI and no further irradiation treatments. He was diagnosed 4 years after treatment and relapsed after another 2 years; the patient succumbed to the disease 16 months later. The second patient developed HD 6 years after 12-Gy cobalt-60 TBI on the ALL-BFM 2000 protocol. The patient also had no further irradiation treatments. He was treated for HD and is alive. The third patient developed rhabdomyosarcoma 6 years after 12-Gy photon TBI. The patient was treated for rhabdomyosarcoma and is alive.

**Table 1:** Patients’ characteristics. Our cohort comprises predominantly male pts. with acute lymphatic leukemia (ALL) in CR1 or 2

Cohort size	*n* = 109
Age	2.4–18.9 years (median 11.2 years)
Sex ratio	34 female: 75 male
Diagnoses	ALL (*n* = 101) other (AML = 5, CML = 2, NHL = 1; *n* = 8)
Initial study protocol	92% of pts. received initial therapy on or according to a current study protocol (ALL-BFM 90, ALL-BFM 95, ALL-REZ BFM 96, ALCL 99, ALL-BFM 2000, COALL 06-97, GMALL 06-99, GMALL 08-03)
Conditioning protocol	EBMT handbook and guidelines, ALL SCTped 2012 FORUM trial (TBI was 6 × 2 Gy b.i. d. q8 h on 3 consecutive days, mean lung dose ~ 10 Gy)
Prior irradiation	*n* = 45 (WBRT *n* = 22; gonadal boost *n* = 10; misc. *n* = 13)

## Discussion

In this cohort of 109 pts. treated with the same TBI regimen as part of their conditioning prior to SCT, tumor control is in keeping with published results, with OS at 5 years of 75.5% and a low recurrence rate of 7.3% (Fig. 2). In this multimodally treated cohort, late effects cannot be attributed to a single therapy element but are a consequence of combined effects. With complete organ-at-risk (OAR) exposure in TBI though, the estimation of radiation-induced late effects is higher than in focal radiotherapy [6].

GD (24%) and hypothyroidism (21.2%) are the most frequent late effects [7], with a median time to onset of hypothyroidism of 50 months [8]. Compared to single-session TBI, fractionated TBI reduces the rate of hypothyroidism from 90 to 15% [9], which is reflected by the 12.5% in our cohort. Young age is conversely associated with an increase in GD rates. In a retrospective analysis of 81 very young pts. (median age 1.4 years), 92% had growth hormone (GH) deficiency with 71% falling under the 2.5% growth percentile [3, 10], while 80% still show a growth > 3rd percentile [11–13]. In our cohort, 31% of pts. with GD had prior WBRT, which increases the dose to the pituitary [14].

Fractionated TBI decreases the cataract rate from > 90% to 30% at 3 years [15]. In our cohort we saw a cataract rate of only 2.7%, which is probably due to fractionation and the higher median age of 11.2 years.

Restrictive lung disease is seen in ~ 20% of pediatric pts. and peaks 3–6 months after TBI/SCT. Restitution is possible within 2 years [16–19]. Thirteen (12%) of our pts. developed cryptogenic organizing pneumonia (COP) with decreased FEV‑1.

The main risk factor for glomerulopathy after TBI/SCT in children is TBI [20]. Grönros et al. analyzed 187 pts. undergoing SCT, 64 pts. had an additional TBI. Fifty healthy children constituted the control group. GFR and effective renal plasma flow were normal prior to therapy. After treatment both values were significantly worse with TBI [21]. A study on 1635 adult pts. (median age at SCT 38.5 years) on the other hand could show that a reduction in GFR post TBI and SCT was mainly associated with GVHD [22]. Seven of our pts. developed renal insufficiency with chronically elevated retention parameters ([3]; Fig. 2).

Rösler and co-workers presented a cohort of 216 pts. with irradiation to the liver (70 pts. with TBI). With a mean dose to the liver of 5 Gy, 17% of pts. developed mild liver dysfunction [23]. With a mean dose of 12 Gy we saw 32 pts. (28.4%) with pathologic serum liver parameters. There was no case of VOD (veno-occlusive disease).

Four pts. in our cohort were diagnosed with dyslipidemia, which is in accordance with two studies showing elevated triglycerides in children after TBI and SCT [24, 25]. Dyslipidemia might be associated with GH deficiency; hence, early GH substitution might normalize serum lipids and thus decrease cardiovascular risk [26, 27].

Secondary malignancies after TBI and SCT are mostly lymphomas (often earlier after SCT) and hematologic malignancies. In most cases they are associated with immune dysregulation and EBV reactivation/infection [28, 29]. Two of our pts. (1.8%) developed Hodgkin lymphoma (HD) in the FU period. Secondary solid tumors are rare but occur with a two- to threefold risk compared to the normal population. They occur after long latency periods. Young age is an independent risk factor. There is a dose dependency, especially in secondary tumors of the thyroid, salivary glands, bone, connective tissue, and brain [30–35]. One of our pts. developed rhabdomyosarcoma 6 years after treatment. In other publications rates of secondary solid tumors were higher [36–38], especially in younger pts. [30].

There are critical issues and shortcomings concerning this analysis. The retrospective nature of this work alone is susceptible to fault. The pts. in this analysis were not systematically screened, but rather we relied on the available FU data. On the other hand, one might argue that only clinically apparent conditions impair a patient’s quality of life and, hence, subclinical deviations could be neglected. The strength and uniqueness of this work is obviously in the large cohort size treated at one institution with the same TBI regimen in combination with a very long FU period. As all pts. were treated with CT and radiation, the adverse events described here result from both modalities, without a possible means of discrimination.

## Conclusion

Tumoricidal effects and immune suppression of TBI facilitating donor cell engraftment are well established. Hence, TBI remains a vital part of conditioning regimens in pts. with risk factors or recurrences prior to SCT and its’ omission can have detrimental outcome effects. Severe long-term effects seem rare when looking at large cohorts with long FU. But individually, they can pose life-altering events, especially in very young pts. Hence, further optimization of techniques and long-term FU programs for the detection of rare events are warranted. We established helical tomotherapy-based intensity-modulated conformal TBI in children and young adults. With this technique we are able to reduce the dose to critical organs such as the eyes [39], the thyroid, the liver, and the lungs [40]. Losert et al. have proposed a rotatable tabletop system to provide a similar treatment on a regular linac [41]. Total marrow irradiation and total marrow and lymphoid irradiation are targeted forms of radiotherapy that have the potential to decrease toxicity [42]. In the murine model, ultra-high-dose-rate FLASH-TBI has been shown to be beneficial with regard to hematopoiesis preservation in leukemia treatment [43].
